# The benefit of complete resection of contrast enhancing tumor in glioblastoma patients: A population-based study

**DOI:** 10.1093/nop/npad037

**Published:** 2023-07-03

**Authors:** Eduardo Erasmo Mendoza Mireles, Erlend Skaga, Andres Server, Henning Leske, Petter Brandal, Eirik Helseth, Pål A Rønning, Einar O Vik-Mo

**Affiliations:** Department of Neurosurgery, Oslo University Hospital, Oslo, Norway; Vilhelm Magnus Laboratory, Institute for Surgical Research, Oslo University Hospital, Oslo, Norway; Department of Neurosurgery, Oslo University Hospital, Oslo, Norway; Vilhelm Magnus Laboratory, Institute for Surgical Research, Oslo University Hospital, Oslo, Norway; Department of Radiology, Oslo University Hospital, Oslo, Norway; Department of Pathology, Oslo University Hospital, Oslo, Norway; Department of Oncology, Oslo University Hospital, Oslo, Norway; Department of Neurosurgery, Oslo University Hospital, Oslo, Norway; Faculty of Medicine, Institute of Clinical Medicine, University of Oslo, Oslo, Norway; Department of Neurosurgery, Oslo University Hospital, Oslo, Norway; Department of Neurosurgery, Oslo University Hospital, Oslo, Norway; Vilhelm Magnus Laboratory, Institute for Surgical Research, Oslo University Hospital, Oslo, Norway; Faculty of Medicine, Institute of Clinical Medicine, University of Oslo, Oslo, Norway

**Keywords:** complete resection of contrast enhanced tumor, extent of resection, glioblastoma, increased survival

## Abstract

**Background:**

New treatment modalities have not been widely adopted for patients with glioblastoma (GBM) after the addition of temozolomide to radiotherapy. We hypothesize that increased extent of resection (EOR) has resulted in improved survival for surgically treated patients with glioblastoma at the population level.

**Methods:**

Retrospective analysis of adult patients operated for glioblastoma in the population of South–Eastern Norway. Patients were stratified into Pre-temozolomide- (2003–2005), temozolomide- (2006–2012), and resection-focused period (2013–2019) and evaluated according to age and EOR.

**Results:**

The study included 1657 adult patients operated on for supratentorial glioblastoma. The incidence of histologically confirmed glioblastoma increased from 3.7 in 2003 to 5.3 per 100 000 in 2019. The median survival was 11.4 months. Complete resection of contrast-enhancing tumor (CRCET) was achieved in 386 patients, and this fraction increased from 13% to 32% across the periods. Significant improvement in median survival was found between the first 2 periods and the last (10.5 and 10.6 vs. 12.3 months; *P* < .01), with a significant increase in 3- and 5-year survival probability to 12% and 6% (*P* < .01). Patients with CRCET survived longer than patients with non-CRCET (16.1 vs. 10.8 months; *P* < .001). The median survival doubled in patients ≥70 years and (12.1 months). Survival was similar between the time periods in patients where CRCET was achieved.

**Conclusions:**

We demonstrate an improved survival of GBM patients at the population level associated with an increased fraction of patients with CRCET. The data support the importance of CRCET to improve glioblastoma patient outcomes.

Glioblastoma (GBM) is the most common malignant primary brain tumor in adults. Current treatment modalities consist of surgery followed by radio- and chemo-therapy with temozolomide (TMZ). Despite multi-modal oncological treatment, the prognosis is poor^1^ as these tumors harbor a range of therapeutic challenges due to their invasive growth, intra- and intertumoral heterogeneity, and the inability of chemotherapeutic drugs to cross the blood-brain barrier.^2,3^ Since the introduction of routine postsurgical radiotherapy in the 1970s^4,5^ only concomitant and adjuvant TMZ has demonstrated improved survival and received wide implementation.^6^ Although studies have found improved survival using alternating tumor-treating fields, this technology has not been adopted widely due to logistical and financial challenges.^7^

Surgery aims for maximal resection of the tumor while preserving neurological function. No formal randomized studies have been performed to determine the effect of complete resection of contrast-enhancing tissue (CRCET) on survival,^8^ and due to apparent lack of equipoise such a study is unlikely to be performed.^9^ A post hoc analysis of the randomized controlled study evaluating the use of 5-ALA for GBM resection,^10^ found CRCET to be a strong predictor of overall survival and is considered the best data to support maximal resection of contrast-enhancing tumor. CRCET has been found to correlate with prolonged survival in a review analyzing several retrospective studies and some prospective randomized trials.^11^ Meta-analyses of these retrospective data found that CRCET can improve the median survival to 15 months,^12,13^ but these analyzes are criticized due to a potentially skewed patient selection.^14,15^ In recent years, reports supporting additional supramarginal resections of GBM have emerged.^16^ Collectively, this has shifted the surgical approach over the last decade towards more comprehensive resections. However, retrospective studies and selected surgical cohorts are prone to selection bias. Whether a more radical surgical approach is beneficial at the population level in a real-world setting of GBM patients has not been reported.

Due to long traditions of carefully maintained patient registries in publicly funded national healthcare systems, the Nordic countries have a unique opportunity for population-based studies.^17^ Since all patients are tracked with social security numbers and treated within the same healthcare system, follow-up and reliability are very high. Through such registries, it has been possible to monitor and analyze survival for glioblastoma patients before and after TMZ introduction. In the Norwegian population, we have previously reported on the increase in median survival at the population level between the pre-TMZ and TMZ-era from 8.3 to 10.1 months.^18^ Similar survival benefits have been demonstrated in the Nordic countries^19–22^ and in other countries using real-world data.^23–25^

We hypothesize that the broadly increased focus on extent of resection (EOR) has improved the frequency of CRCET, increasing, at the same time, the overall survival of patients operated for GBM between 2003 and 2019.

## Materials and Methods

### Population

Inhabitants of Norway are registered in The National Population Register with a unique ID number and contact information that facilitates contact between health care officials and individual patients. Oslo University Hospital (OUH), consists of 2 neurosurgical centers, which are the only referral centers for neurosurgery in the South–Eastern Norwegian Health Region, with a total population of 3.2 million (55% of the Norwegian population). The demographic distribution of the population has changed little over time and the population ≥ 70 years has remained stable accounting for 15% of the adult population.

### Death Registry

When a patient dies in Norway, the death certificate is sent to both the district court and the local police. The district court will notify the National Registry of the death. Our electronic journal is regularly updated with information from the National Registry of the Death. The final date of data collection were January 01, 2023. All patients that were alive at the time of censoring, were checked for electronic journal updates. Patients that did not have entries in 2022 were contacted directly or through the local hospital to verify they were still alive, and thus reducing to the minimum number of patients that were actually lost to follow-up.

### Patient population

In this study, we identified all patients diagnosed with GBM from 2003 to 2019 (*n* = 1665). The adult population in 2003 and 2019 was 1 944 813 and 2 379 381 inhabitants, respectively. All patients operated on for brain tumors were prospectively registered since 2003 in an OUH institutional quality-control database. We have included all first-time operated adult patients (≥18 years) for histopathologically verified supratentorial GBM (2003–2016), GBM WHO grade IV (2016–2019), as well as tumors classified as gliosarcoma, giant cell GBM, or epithelioid GBM. Patients that underwent a surgical procedure of either biopsy, subtotal or complete resections were included. Patients with prior (before 2003) surgery for GBM or other gliomas, infratentorial localization, and patients with incomplete basic data (national ID number, birth date, and gender) were excluded.

### Definition of Time Periods

Since TMZ as standard-of-care did not receive wide implementation before 2005, we defined the years 2003–2005 as the pre-TMZ period. The interval between 2006 and 2012, in which TMZ became widely adopted as the standard of care, was defined the TMZ period. The shift towards more comprehensive tumor resections over the last decade defined the last period (2013-2019) as the resection-focused period.

### Evaluation of Extent of Resection

A T1 MRI with contrast-enhanced images, either in 3D volume series, or in 3 planes pre- and postoperatively within 72 hours was performed in all patients not having a contraindication to MRI (ie, non-MRI compatible pacemaker). When MRI was contraindicated, a contrast-enhanced CT scan was performed. EOR was evaluated according to incomplete- or complete resection of all contrast-enhanced tumors. Resection was classified as subtotal when there was any sign of contrast-enhancing remnants in the MRI or CT scan, when patients had other lesions that were not addressed surgically, or when the surgical report clearly expressed that not all tumor was removed. In cases of imaging ambiguity, resection was registered as incomplete. When there was not enough data it was ruled as not specified, to avoid inaccuracies. Molecular analysis of *IDH* and *MGMT* were sporadic until the end of the last period and thus not included in the study analysis. For missing data, files were updated from a review of the patient’s electronic journal.

### Statistical Analysis

We calculated age-specific glioblastoma incidence per 100 000 inhabitants using population statistics from The Official Statistical Bureau of Norway (Statistic Norway).^26^ Population is reported by gender, age, and district. Incidence is calculated overall and by age group. Overall survival analysis was used to compare resection grades and relative survival to compare period groups. Relative survival, the cancer survival in the absence of other causes of death, was calculated according to the Pohar-Perme method^27^ and data from The Human Mortality Database using the relsurv package.^28^ Reverse Kaplan–Meier was used to calculating median follow-up.^29^ Chi-square and log-rank tests were used to compare groups. Comparisonsurv package was used to calculate survival probability at 2-, 3- and 5-years, and logarithmic conversion as described by Klein et al.^30^ was used to calculate its p-value. Uni- and multivariable Cox regression was used to generate hazard ratios for censored variables after verifying that the model fulfilled assumptions of proportional hazard. Bonferroni correction was performed to correct for multiple testing. Patients were evaluated according to treatment periods, age groups, age at the time of first operation (18–45, 46–69, and ≥70 years old), affected lobe, gender, operation type (biopsy or resection), and EOR (subtotal resection, complete resection of enhanced contrast tumor, and not available). All analyses were performed using R Statistical Software (v4.1.2; R Core Team 2021).

### Ethical Consideration

The study was approved by OUS Data Protection Officer and the South–Eastern Regional Ethical Committee (21/02197).

## Results

Between 2003 and 2019, a total of 1665 patients with a biopsy-confirmed diagnosis of GBM were treated at OUH. Eight patients were excluded, either due to emigration within a month after primary surgery (*n* = 6), or were non-citizens (without national identification number, *n* = 2). This resulted in a total of 1657 patients included in this study. Just 3 patients were lost at follow-up and were censored based on their last journal entry.

### Population Overview

**Figure 1.**
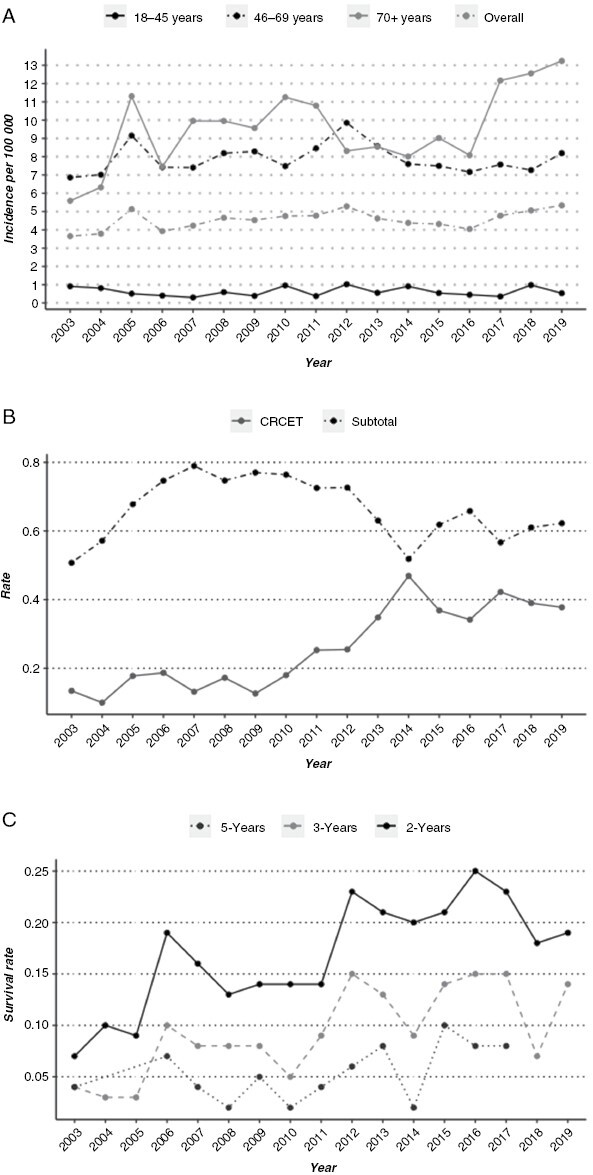
(A) Incidence of patients operated on (biopsy and resection) for glioblastoma analyzed according to year of surgery and age group. (B) Rate of complete resection of contrast-enhancing tumor (CRCET) and subtotal resection analyzed according to year of surgery. (C) Survival rate of 2- and 5-year survival analyzed according to year of surgery.

The median age at diagnosis was 64 (interquartile range 55–71) years and there was a slight male predominance (1.44:1). The incidence of histologically verified GBM increased from 3.7 per 100 000 inhabitants in 2003 to 5.3 per 100 000 inhabitants in 2019 (Figure 1A). In the youngest patient group (18–45 years), the incidence was constant during the different study periods. In contrast, the incidence increased in the oldest age group (≥70 years) from 5.6 per 100 000 in 2003 to 13.2 per 100 000 in 2019 (Figure 1A). Evaluation of the EOR demonstrated that more patients received CRCET in the resection-focused period (2013–2019, 32%) compared to the 2 previous (13% and 17%, respectively, Table 1 and Figure 1B). Over the study period the median, 2-, 3-, and 5-year survival gradually improved (Figure 1C). The 30-day mortality was reduced from 4% to 2% over the time periods, with a total 30-day mortality across the entire study period of 3%. Reoperation at the time of tumor recurrence was performed in around 12% of patients and remained constant during the study period. Further patient characteristics are outlined in Table 1. Importantly, the incidence of resection and biopsies were similar over the last 2 periods (*P* = .66). Median follow-up was 105 months.

**Table 1.** Patients´ and Tumor´s Characteristics

Characteristic	2003–2005, *N* = 243	2006–2012, *N* = 668	2013–2019, *N* = 746	Overall, *N* = 1657
**Age in years**				
**Median (IQR)**^a^	63 (55, 70)	63 (56, 70)	64 (54, 71)	64 (55, 71)
**Gender**				
Female	100 (41%)	277 (41%)	300 (40%)	677 (41%)
Male	143 (59%)	391 (59%)	446 (60%)	980 (59%)
**Age group**				
18–45	21 (8.6%)	41 (6.1%)	48 (6.4%)	110 (6.6%)
46–69	157 (65%)	436 (65%)	462 (62%)	1,055 (64%)
70+	65 (27%)	191 (29%)	236 (32%)	492 (30%)
**Lobe**				
Frontal	66 (27%)	214 (32%)	253 (34%)	533 (32%)
Temporal	68 (28%)	176 (26%)	216 (29%)	460 (28%)
Parietal	42 (17%)	104 (16%)	123 (16%)	269 (16%)
Two or more regions	32 (13%)	80 (12%)	76 (10%)	188 (11%)
Occipital	24 (9.9%)	66 (9.9%)	47 (6.3%)	137 (8.3%)
Corpus Callosum	8 (3.3%)	17 (2.5%)	20 (2.7%)	45 (2.7%)
Insula	3 (1.2%)	11 (1.6%)	11 (1.5%)	25 (1.5%)
**Side**				
Right	124 (51%)	315 (47%)	371 (50%)	810 (49%)
Left	107 (44%)	317 (47%)	346 (46%)	770 (46%)
Bilateral	9 (3.7%)	22 (3.3%)	21 (2.8%)	52 (3.1%)
Midline	3 (1.2%)	14 (2.1%)	8 (1.1%)	25 (1.5%)
**Extent of resection**				
Subtotal	134 (55%)	459 (69%)	372 (50%)	965 (58%)
CRCET	32 (13%)	115 (17%)	239 (32%)	386 (23%)
Biopsy	18 (7.4%)	58 (8.7%)	130 (17%)	206 (12%)
Not Classified EOR	59 (24%)	36 (5.4%)	5 (0.7%)	100 (6.0%)
**30 days mortality**	10 (4.1%)	25 (3.7%)	19 (2.5%)	54 (3.3%)
**Reoperation**	29 (12%)	91 (14%)	96 (13%)	216 (13%)

^a^ Interquartile range.

### Survival Between the Time Periods

**Figure 2.**
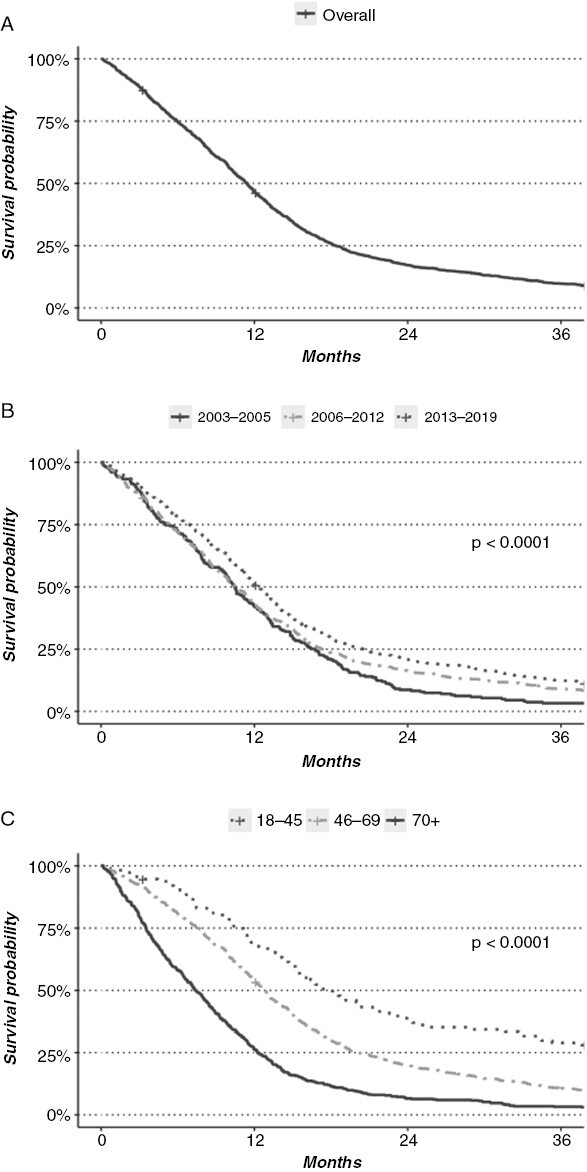
(A) Overall survival of 1657 patients with glioblastoma undergoing surgery. (B) Survival analyzed according to time period. (C) Survival analyzed according to age group.

The median survival across all time periods was 11.4 months (95% CI 10.8–11.8, Figure 2A). The 1-, 2-, and 5-year survival rates were 47%, 17%, and 5%, respectively. We found an improved median survival from 10.5 months (95% CI 9.6–11.8) in the pre-TMZ period to 12.3 (95% CI 11.6–13.1) months in the resection-focused period (*P* < .01). Similarly, a higher fraction of patients was alive 2 years after surgery (from 17% to 21%, *P* < .05, Figure 2B). The 5-year survival probability was compared for the first 2 groups, as this was not reached for latest period, where we found an improvement between the pre-TMZ (1%) and the TMZ period (4%, *P* < .01). Younger patients demonstrated better median survival compared to patients ≥ 70 years (18.1 vs. 7.4, *P* < .001, Figure 2C).

### Extent of Resection

**Figure 3.**
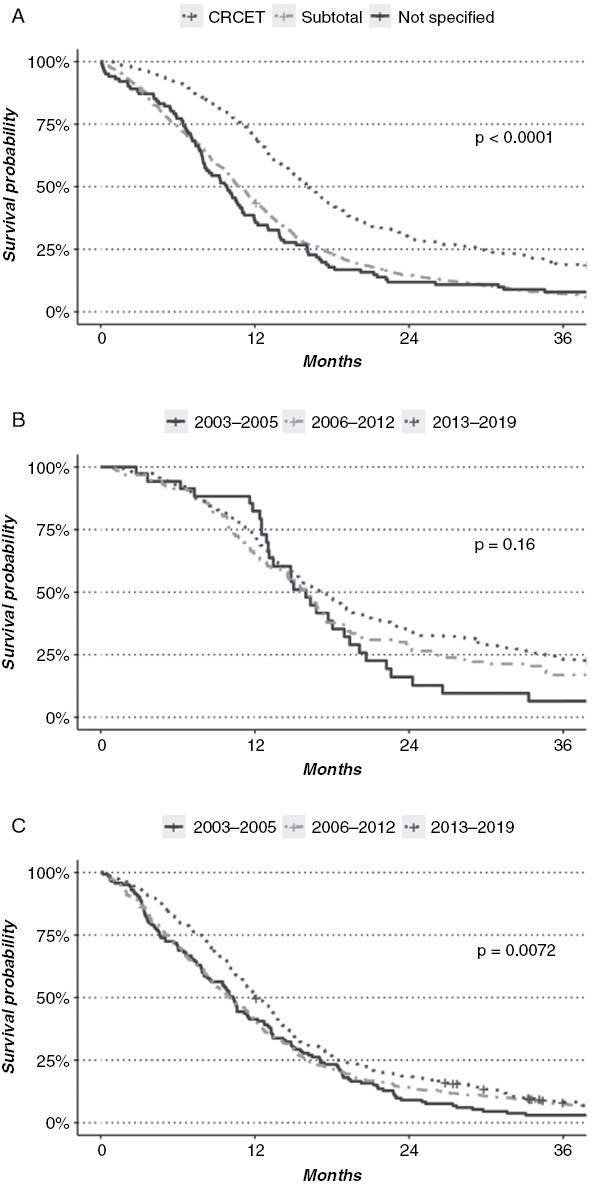
(A) Overall survival of 1451 patients with glioblastoma undergoing resection analyzed according to extent of resection (CRCET; complete resection of contrast-enhancing tumor). (B) Overall survival of 386 patients with glioblastoma where CRCET was achieved analyzed according to time period. (C) Overall survival of 965 patients with glioblastoma where subtotal resection of contrast-enhancing tumor was achieved analyzed according to time period.

Patients with CRCET had a median survival of 16.1 months (95% CI 15.1–17.5), whereas patients with a subtotal resection had a median survival of 10.8 months (*P* = .001, Figure 3A). In 100 patients undergoing craniotomy with tumor resection, we were unable to assess the postoperative resection grade (Figure 3A). In this latter group, the median survival was 9.8 months, insignificantly different from the subtotal resection group (*P* = .76). The rate of CRCET gradually improved over the years (Figure 1B), with increasing percentage over the time periods (13%, 17%, and 32%). When comparing the median survival in patients where CRCET was achieved, we found similar survival between the 3 time periods (Figure 3B), 15.0 months (95% CI; 13.1–19.4) for the pre-TMZ period, 15.5 months (95% CI; 14.0–17.0, *P* = .81) for the TMZ period, and 16.7 months (95% CI; 15.1–18.9, *P* = .1) for the resection-focused period. When comparing the median survival of patients that underwent subtotal resection by period, we found a statistically significant survival benefit in the latter time period, 10.3 months versus 10.0 months versus 12 months, in the different periods respectively (*P* < .01, Figure 3C).

We further evaluated the effects of age and grade of resection combined. The median survival for the older age group (≥70 years) was 7.4 months (95%CI 6.5–8.1), 12.8 months (*P* < .001, 95% CI 12.1–13.3) for patients between 46 and 69 years, and 18.1 months for patients in the 18–45 group (*P* < .001; 95%CI 15.4–22.8; Figure 2C). Comparing CRCET and subtotal resection for each age group, the median survival increased from 15.6 to 35.3 months in the younger group, from 11.4 to 16.8 months for patients 46–69 years, and from 6.3 to 12.1 months in patients ≥ 70 years (all *P* < .01).

### Cox Multivariable Analysis

The multivariable analysis overall supported the findings above (Table 2). There was no effect of tumor laterality nor gender. We found a decrease in mortality risk over time, with 18% risk reduction between pre-TMZ and TMZ periods (*P *= .016), and a 35% risk reduction between the post-TMZ and resection-focused period (*P* < .001). Patients with subtotal resection had a 63% increased mortality risk (*P* < .001), while patients, where only a biopsy was performed, had a 178% increased risk compared to the CRCET group (p < 0.001).

**Table 2.** Cox Uni- and Multi-variable Regression Analysis

Characteristic	Univariable			Multivariable		
	HR	95% CI	*P*-value	HR	95% CI	*P*-value
**Age**						
18–45	—	—		—	—	
46–69	1.70	1.38, 2.10	<.001	1.83	1.46, 2.29	<.001
70+	3.26	2.61, 4.06	<.001	3.86	3.04, 4.92	<.001
**Period**						
2003–2005	—	—		—	—	
2006–2012	0.85	0.73, 0.98	.030	0.82	0.70, 0.97	0.024
2013–2019	0.71	0.61, 0.83	<.001	0.70	0.59, 0.82	<.001
**Gender**						
Female	—	—		—	—	
Male	1.09	0.99, 1.21	.087	1.09	0.98, 1.22	.12
**Lobe**						
Frontal	—	—		—	—	
Parietal	1.00	0.86, 1.16	>.9	0.99	0.84, 1.16	.9
Temporal	1.02	0.89, 1.15	.8	1.01	0.88, 1.15	>.9
Occipital	1.32	1.09, 1.59	.004	1.15	0.94, 1.41	.2
Insula	1.31	0.87, 1.95	.2	1.35	0.89, 2.05	.2
Corpus Callosum	1.60	1.18, 2.17	.003	1.41	0.95, 2.10	.090
Two or more regions	1.85	1.56, 2.19	<.001	1.65	1.36, 1.99	<.001
**Side**						
Right	—	—		—	—	
Left	1.00	0.91, 1.11	>.9	1.08	0.97, 1.20	.2
Midline	1.46	0.98, 2.18	.060	1.06	0.64, 1.75	.8
Bilateral	2.13	1.61, 2.82	<.001	1.35	1.00, 1.82	.053
**EOR**						
CRCET	—	—		—	—	
Subtotal	1.74	1.54, 1.97	<.001	1.68	1.47, 1.92	<.001
Biopsy	3.30	2.77, 3.93	<.001	2.90	2.38, 3.53	<.001

## Discussion

The data are in line with several retrospective surgical cohorts suggesting that EOR is associated with improved patient survival.^31,32^ No randomized controlled study exists that establishes this association,^33,34^ and retrospective studies are prone to selection bias. The presented data should be less prone to such skewness, as all patients within the region are treated at this institution and loss to follow-up is limited. This reduces the risk of patient selection bias and improves external validity. There was a nonsignificant increase in both the biopsy and resection incidence during the last period, excluding the possibility that the increase in CRCET and survival was caused by a more restrictive patient selection. We found that the incidence of histologically verified GBM increased over time, especially in the older population (≥70 years). That patients undergoing biopsy had a dismal prognosis, and that CRCET increased median survival. The benefit of CRCET was present within all age groups. We further found a statistically significant increase in the median, 2-, and 5-year survival over time. Specifically evaluating only patients with CRCET, we found similar median survival over all time periods. Overall, these real-world data support the impact of CRCET on improving patient prognosis in glioblastoma patients at a population level across all age groups.

The improved EOR over time is likely confounded by the introduction of more advanced imaging, intraoperative supportive tools, and closer follow-up. The combination of these efforts may contribute to the improved outcome over time. During the last period, the Norwegian Health Care system also introduced defined clinical care pathways that guarantee patients with GBM a planned and timely treatment coordination.^35^ Such clinical care pathways could also have contributed to the improved survival in the resection-focused period. In support of clinical care pathways, we found improved survival in the last period and also in patients not receiving complete resection.

Several studies have addressed EOR and its impact on survival. Woo et al,^36^ evaluated 1010 patients operated on between 2006 and 2019, and Koshy et al.^37^ evaluated 6919 patients operated on between 2000 and 2006. Although both studies found improvement in survival in patients receiving gross total resection, the EOR was based on the surgeon´s preoperative evaluation and not on the postoperative contrast enhancement residual. Scoccianti et al.^38^ included 1059 patients in an Italian multicenter study. They found significantly increased survival in patients where no residual contrast enhancement was seen on postoperative CT or MRI, but also included patients based on surgical reports when no postoperative imaging was available. Pan et al., evaluated 14 560 patients, covering an estimated 26% of the US population. EOR was divided into gross total resection, partial resection, and biopsy, but no clear definition of gross total resection was described.^39^ Thus, while all of these studies support the importance of optimized resection, they do not do so consistently based on postoperative images. In the current study, postoperative imaging within 72 hours was used to classify the resection. CRCET was scored only when there were no remaining contrast-enhancing volumes, corresponding to class 2a in the recent RANO resect criteria.^32^

While the improvement in median survival was largest between the last 2 periods, the improvement in long-term survivors is largest between the 2 first periods. This could suggest that the increased CRCET fraction primarily affects median survival and early tumor recurrence, while the introduction of TMZ is the main determinant for long-term survivorship. This is compatible with other studies,^16,32^ suggesting that the impact of resection is tied to the removal of the most rapidly proliferating part of the tumor.

The presented study has several limitations. Although this study comprehends 2 centers, they belong to a same institution, and thus could reflect changes specific to this institution. The previous studies from our institution^18,22^ have found similar survival estimates to other population-based studies when evaluating the introduction of TMZ,^19–21,24,40^ seems to suggest that this effect is moderate. Recent studies have suggested a classification of EOR,^41–43^ but as the study database have been prospectively curated we have just classified EOR as complete or incomplete based on contrast-enhancing tumor. Molecular data on *IDH* mutation status and *MGMT*-promotor methylation were not regularly evaluated in the first half of the study period and have therefore been omitted from the analysis. We acknowledge the integral part those molecular markers represent in current diagnostics and in prediction of response to treatment. Furthermore, individualized data on radiotherapy or TMZ treatment is not included in the current registry, but further studies should include these parameters for a more thorough evaluation.

## Conclusion

This is the first study to demonstrate, at a population level, that increased CRCET improves survival in patients with GBM. Between 2003 and 2019, patients operated on for glioblastoma lived longer. This survival improvement is correlated to the introduction of combined radio- and chemotherapy (from the first to second period) and more patients receiving CRCET (from the second to third period). The benefit in survival in the group of patients undergoing CRCET is consistent over time and spans all age groups, also patients 70 years or older. Collectively, this further suggests a more comprehensive surgical approach to treat GBM, not only in the younger patients, but also in the group of patients of advanced age. Further studies to analyze the effect of molecular data, formalized evaluation of EOR, and the impact of patient care pathways may increase our understanding of the current treatment given further.

## Data Availability

The data that support the findings of this study are available from the corresponding author, EEMM, upon reasonable request.
